# Severe length‐dependent peripheral polyneuropathy in a patient with subacute combined spinal cord degeneration secondary to recreational nitrous oxide abuse: A case report and literature review

**DOI:** 10.1002/ccr3.6881

**Published:** 2023-02-13

**Authors:** Ventzislav Bonev, Mark Wyatt, Matthew J. Barton, Michael A. Leitch

**Affiliations:** ^1^ School of Medicine Griffith University Gold Coast Queensland Australia; ^2^ Coastal Neurophysiology Services Gold Coast Queensland Australia; ^3^ School of Nursing and Midwifery Griffith University Gold Coast Queensland Australia; ^4^ Menzies Health Institute Queensland Gold Coast Queensland Australia; ^5^ Neurosciences Queensland Brisbane Queensland Australia

**Keywords:** myelopathy, nanging, nitrous oxide, polyneuropathy

## Abstract

Nitrous oxide abuse can have detrimental effects on the central and peripheral nervous systems. This case study report aims to demonstrate a combination of severe generalized sensorimotor polyneuropathy and cervical myelopathy related to vitamin B_12_ deficiency following nitrous oxide abuse. We present a clinical case study and literature review examining primary research—published between 2012 and 2022—reporting nitrous oxide abuse affecting the spinal cord (myelopathy) and peripheral nerves (polyneuropathy); 35 articles were included in the review with a total of 96 patients, where the mean “patients” age was 23.9 years and were in a 2:1 male/female ratio. Of the 96 cases, within the review, 56% of patients were diagnosed with polyneuropathy, most commonly impacting the nerves of the lower limb (62%), while 70% of patients were diagnosed with myelopathy, most commonly impacting the cervical region (78%) on the spinal cord. In our clinical case study, a 28‐year‐old male underwent a multitude of diagnostic investigations for bilateral “foot drop” and sense of lower limb stiffness as ongoing complications of a vitamin B_12_ deficiency secondary to recreational nitrous oxide abuse. Both the literature review and our case report emphasize the dangers of recreational nitrous oxide inhalation, colloquially termed “*nanging*” and the risks it presents to both the central and peripheral nervous systems, which is erroneously considered by many recreational drug users to be less harmful than other illicit substances.

## Abstract 2

Our case report emphasizes the danger of recreational nitrous oxide inhalation and the risks it presents to both the central and peripheral nervous systems, which is considered by many recreational users to be less harmful than other illicit substances.

## 1 BACKGROUND

Nitrous oxide (N_2_O), colloquially known as “laughing gas,” has increasingly begun to be used as a recreational drug in Australia and other countries worldwide, referred to by users as “*nanging*.” Given how cheap and readily available N_2_O is to the general public and that recreational drug users consider that “*nanging*” is a harmless alternative to other drugs, it is important that the recreational use of N_2_O is limited, and the potential users are alerted and educated to the serious and long‐term consequences of this substance.^1^ One of the most clinically significant and adverse effects of N_2_O abuse is vitamin B_12_ deficiency, which can result in peripheral neuropathy and myelopathy.^2^

## 2 CASE REPORT

**FIGURE 1:**
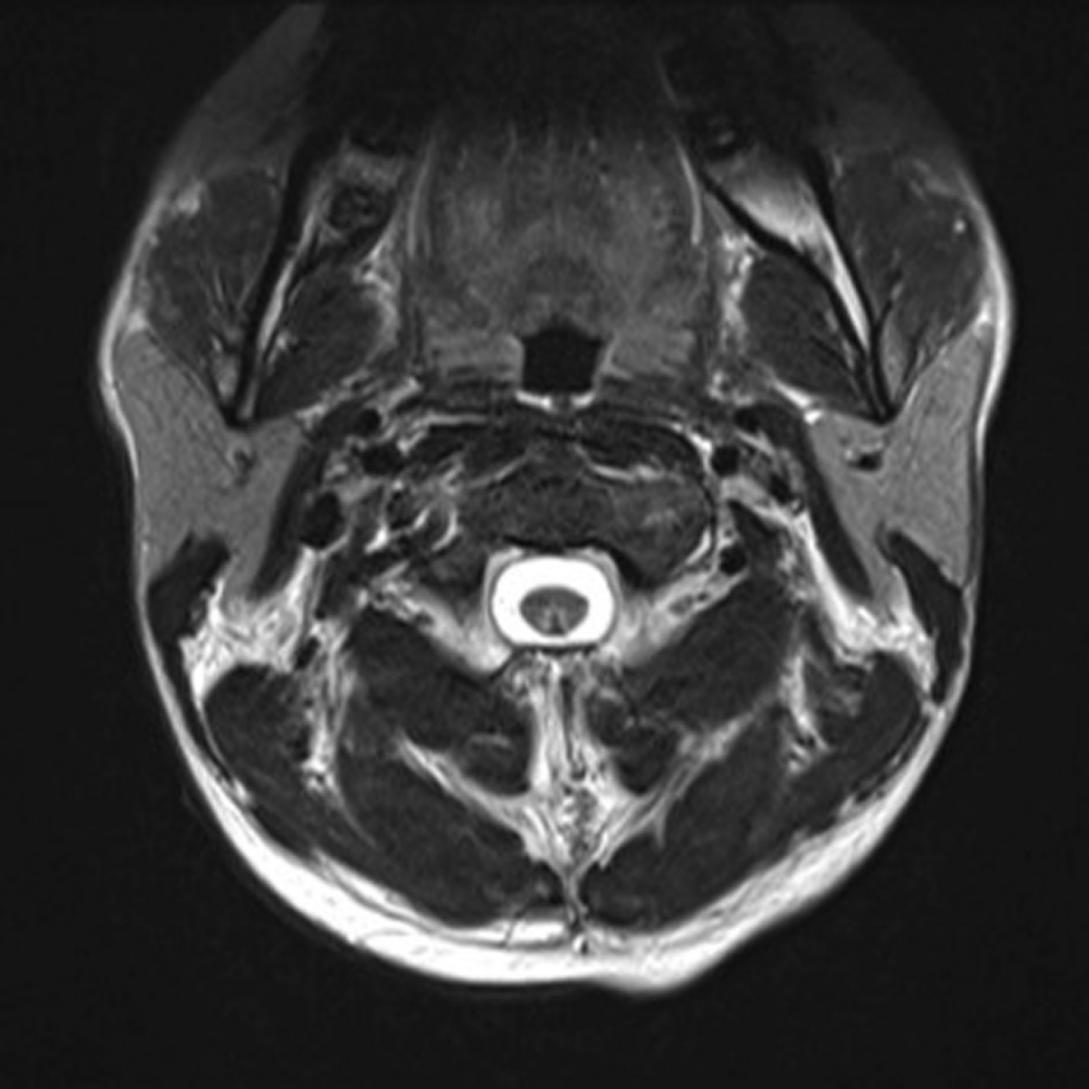
MRI axial T2‐weighted sequences of the cervical region, showing increased signal on the axial T2‐weighted sequence in the posterior columns at C2 and C3.

A 28‐year‐old male was referred by his general practitioner (GP) with a 9‐month history of persistent fatigue, paraesthesia, weakness, and a sense of stiffness in the muscles of the legs and feet. These symptoms commenced after a 3‐month period of substantial recreational N_2_O abuse during which the patient was consuming 80–100 “*nangs*” per day while attempting to cease smoking. The patient's sensory and motor symptoms were first detected after the patient presented to Hospital with septic shock due to pyelonephritis. During this hospital presentation, the patient exhibited upper motor neuron signs in his upper and lower limbs, which included myoclonus, clonus, hyperreflexia and proximal weakness, more specifically, weakness of hip flexion, and ankle plantar flexion. After hospitalization, initial vitamin B_12_ levels were 60 pmol/L (ref.range > 150 pmol/L), this was after 2 × 1 mg vitamin B_12_ injections; patient was anemic with hemoglobin values of 78 g/L (ref.range 135–180 g/L), hematocrit of 0.22 (ref.range 0.39–0.52), and red cell count of 2.01 (ref.range 4.50–6.00 × 10^12^/L). MRI of the brain and entire spinal cord showed increased signal on the axial T2‐weighted sequence in the posterior columns at C2 and C3 (see Figure 1). The patient was diagnosed as suffering from subacute combined spinal cord degeneration secondary to vitamin B_12_ deficiency caused by N_2_O abuse. The patient was admitted and treated for pyelonephritis with antibiotics and with intramuscular vitamin B_12_ injections, including oral methionine. The patient's neurological symptoms improved markedly during his nine‐day admission and subsequently returned to the care of his GP who continued vitamin B_12_ injections every 2 months, and referred him for physiotherapy, and neurophysiological assessment (5–6 months after his hospital presentation). Upon examination at his neurophysiological assessment, he had severe weakness of ankle dorsiflexion, eversion, and toes extension bilaterally, with sparing of ankle plantar flexion, inversion, and toes flexion bilaterally. More proximal examination of the knee and hip girdle muscles was normal, as was the upper‐limb motor examination bilaterally. Ankle deep tendon reflexes were absent bilaterally, knee reflexes were symmetrically increased with crossed adductor reflex present bilaterally. Upper‐limb deep tendon reflexes were normal bilaterally. No clonus or myoclonus was present and no Babinski sign was present on either side. Hoffman's sign was present bilaterally. Cutaneous sensation for pain using a Wartenberg pinwheel was normal. Vibration sense was absent at the toes bilaterally using a 128 Hz tuning fork but was present at the ankles and knees. Gait was markedly impaired from bilateral foot drop with only minimal ataxia. Relevant past medical history included previous cervical spinal surgery of C4/5 fusion and disc replacement, which was performed unremarkably with no sequelae. Of note, the patient continues to smoke tobacco and cannabis and takes un‐prescribed diazepam. Nerve conduction studies assessed sural sensory nerves, peroneal and tibial motor nerves, and tibial H‐reflexes bilaterally. No compound motor action potentials or sensory nerve action potentials could be recorded from the lower limbs (see Tables 1 and 2). Right median and ulnar sensory nerves and right median motor nerve were then assessed, displaying normal onset latency at the wrist and normal nerve‐conduction velocity in the forearm segment with mildly reduced amplitude (see Tables 1 and 2). These findings are consistent with severe, length‐dependent generalized peripheral neuropathy. Differential diagnoses would include metabolic imbalances, genetic disorders, and toxicities; however, detailed history, blood screening, and diagnostics effectively excluded these possibilities. Differential diagnoses of his UMN signs would include cervical myelopathy related to his previous C4/5 disc replacement surgery; however, this was not suggestive, based on his clinical examination or cervical neuroimaging findings.

**TABLE 1:** Sensory nerve conduction studies

Stim Site	Peak (ms)	Norm Peak (ms)	O‐P Amp (μV)	Norm O‐P Amp	Site1	Site2	Delta‐P (ms)	Dist (cm)	Vel (m/s)	Norm Vel (m/s)
Right Superficial Peroneal Anti‐Sensory (Lateral Malleolus)										
Leg	*NR*	<4.2	*NR*	>4	Leg	Lateral Malleolus		14.0		
Right Sural Anti‐Sensory (Lateral Malleolus)										
Calf	*NR*	<4.5	*NR*	>4	Calf	Lateral Malleolus		14.0		
Left Superficial Peroneal Anti‐Sensory (Lateral Malleolus)										
Leg	*NR*	<4.2	*NR*	>4	Leg	Lateral Malleolus		14.0		
Left Sural Anti‐Sensory (Lateral Malleolus)										
Calf	*NR*	<4.5	*NR*	>4	Calf	Lateral Malleolus		14.0		
Right Median Anti‐Sensory (digit II)										
Calf	3.5	<4.0	15.1	>10	Calf	Lateral Malleolus		14.0		
Right Ulnar Anti‐Sensory (digit V)										
Calf	3.3	<4.0	22.7	>6	Calf	Lateral Malleolus		14.0		

**TABLE 2:** Motor nerve conduction studies

Stim Site	Onset (ms)	Norm Onset (ms)	O‐P Amp (mV)	Norm O‐P Amp	Site1	Site2	Delta‐0 (ms)	Dist (cm)	Vel (m/s)	Norm Vel (m/s)
Right Deep Peroneal (EDB) Motor (Extensor Digitorum Brevis)										
Ankle	*NR*	<6.5	*NR*	>2.6						
Right Median (APB) Motor (Abductor Pollicis Brevis)										
Wrist	3.4	<4.6	5.1	>5.9	Wrist	Elbow	4.1	22.0	54	>49
Elbow	7.5		5.0							
Right Tibial (AH) Motor (Abductor Hallucis)										
Ankle	*NR*	<6.1	*NR*	>5.8						
Left Deep Peroneal (EDB) Motor (Extensor Digitorum Brevis)										
Ankle	*NR*	<6.5	*NR*	>2.6						
Left Tibial (AH) Motor (Abductor Hallucis)										
Ankle	*NR*	<6.1	*NR*	>5.8						

Abbreviations: Amp, amplitude; *NR*, no response; O‐P, onset‐peak; Vel, velocity.

## 3 LITERATURE REVIEW

### 3.1 Methods

A comprehensive literature search was done on Medline, Embase, and PubMed databases. Search and MeSH terms for nitrous oxide (N_2_O), myelopathy, and polyneuropathies were identified through preliminary searches and combined with the Boolean terms as follows:

(“nitrous oxide” OR “nanging” OR “laughing gas”) AND (“myelopathy” OR “polyneuropathy”). The search was run on years of coverage from 2012 to the present, and other search filters include human studies, English text only, full text, peer‐reviewed articles, and primary research articles.

### 3.2 Results

**FIGURE 2:**
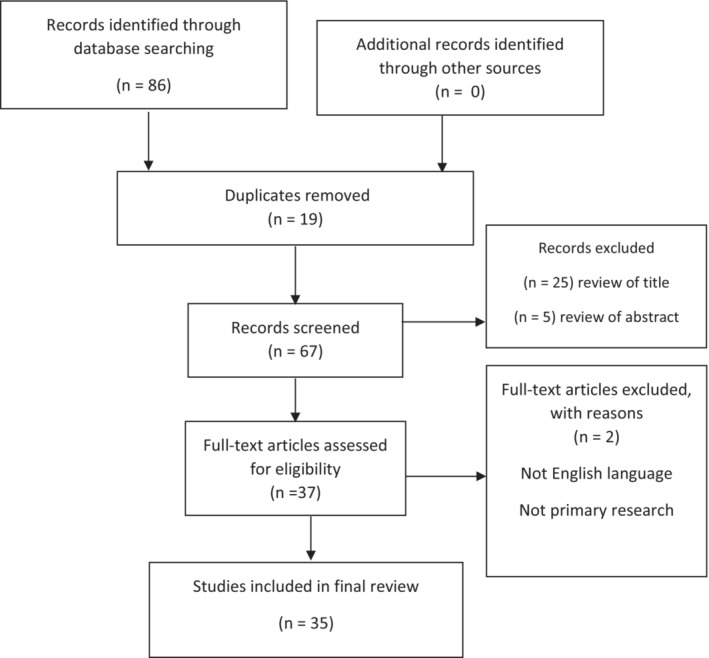
PRISMA flowchart. PRISMA, Preferred Reporting Items for Systematic Reviews and Meta‐Analyses.

Electronic searching identified 86 citations in Medline Ovid, PubMed, and EMBASE from 2012 to 2022. In all, 19 duplicate papers were removed. The remaining papers (*n* = 67) were reviewed for relevance to the topic by title and abstract, with 37 articles meeting the criteria. Full texts with primary research in the English language were subsequently sourced for all articles, with a further two texts excluded. A summary is included using the Preferred Reporting Items for Systematic Reviews and Meta‐Analyses flowchart (Figure 2).

In all, 35 case study articles were included, published between 2012 and 2022, comprising a total of 96 patients. One in three articles were published in the United States, five articles in China, four articles in the UK, and three articles each from Belgium, France, and Taiwan. The “patients” ages ranged from 17 to 50 years of age, with a mean age of 23.9 years and affecting males at a 2:1 male/female ratio.

The patients' neural structures that were affected are summarized in Table 3. The cervical spinal cord (myelopathy) was the most affected neuronal structure, which was seen in 54% of the cases, other regions of the spinal cord affected were thoracic (15%), while polyneuropathy was seen in 53% of cases. Of the peripheral neuropathy cases, the tibial and fibular nerves were affected in 35% of cases, followed by the median (29%), ulnar (27%), and sural (15%) nerves. Further “patients” characteristics are summarized in Table 3. The most common patients' presentations saw 58% of the patients complaining of numbness, just over half of the patients (53%) suffered weakness, difficulty walking (49%), and paraesthesia (40%). Invariably, treatment included ceasing N_2_O use and supplementary therapy of vitamin B_12_ injections. In 45% of patients, after a short period (a couple of weeks) of this regime, saw the partial resolution of neurological symptoms and one‐third of patients fully recovered. Only two patients saw no symptomatic improvement, and this was contributed by the lack of adherence to the treatment regime.

**TABLE 3:** Literature review summary evaluation table

Reference	Country	Patient age/sex	Neuronal structures affected	Symptoms	Outcomes
Li Y et al. 2021^3^	China	22 M; 20 M; 24 M; 18 M; 16 M; 22 F; 21 M; 17 F; 31 F; 22 M; 18 F; 29 M; 21 F; 20 M; 22 M; 26 M; 16 M; 23 M	*n* = 3 cervical spinal cord, *n* = 1 thoracic spinal cord, polyneuropathy affecting (*n* = 16 FN, *n* = 14 TN, *n* = 11 MN, *n* = 11 UN & *n* = 5 SN)	*n* = 9 paraesthesia, *n* = 11 weakness, *n* = 11 numbness, *n* = 4 difficulty walking, *n* = 2 fall/balance difficulty & *n* = 1 foot drop	Not reported
Hirvioja J et al. 2016^4^	Finland	23 Male	axonal motor & mild demyelinating polyneuropathy (lower extremities)	Gait difficulties & numbness	Died (different cause)
Beal JC et al. 2020^11^	USA	17 Female	severe axonal sensorimotor polyneuropathy (TN, PN, MN, and UN), thoracic spinal cord	Weakness, areflexia and numbness	Regained strength and sensation over a period of weeks
Hsu C et al. 2012^12^	Taiwan	19 Male	Cervical spinal cord, sensorimotor polyneuropathy (MN, UN, SN, PN, and TN)	Numbness, weakness and gait imbalance	At 2 months, recovered fully after of supplementation treatment and N_2_O abstinence
Smith CM et al. 2021^5^	USA	26 Male	Distal axonal sensorimotor polyneuropathy	Numbness, weakness and ataxia	Reduction in symptoms at 4‐month follow‐up
Einsiedler M et al. 2022^6^	France	19 M; 20 M; 29 M; 23 F & 27 F	*n* = 3 cervical spinal cord, *n* = 1 demyelinating polyneuropathy, *n* = 1 axonal polyneuropathy	*n* = 4 paraesthesia, *n* = 1 weakness, *n* = 4 numbness, *n* = 2 ataxia	All patients clinical improvement was observed after supplementation therapy
Zhao B et al. 2020^7^	China	21 M & 18 F	*n* = 1 cervical spinal cord, *n* = 1 thoracic spinal cord, *n* = 2 sensorimotor polyneuropathy	*n* = 2 numbness, *n* = 2 weakness, *n* = 1 walking difficulties & *n* = 1 paraesthesia	All patients clinical improvement after supplementation therapy
Berling, E et al 2022^8^	France	20 M; 20 M; 30 M; 18 M; 19 F; 20 M & 19 M	*n* = 2 cervical spinal cord, *n* = 1 thoracic spinal cord, *n* = 6 motor axonal polyneuropathy of the lower limbs	*n* = 6 ataxia, *n* = 7 weakness, *n* = 4 paraesthesia & *n* = 2 numbness.	All patients clinical improvement at short‐term follow‐up after N_2_O discontinuation and supplementation therapy.
Porruvecchio E et al 2022^9^	UK	21 Male	Cervical spinal cord	Weakness, paraesthesia & gait difficulties	Not reported
Choi C et al 2019^10^	Korea	24 M & 22 F	*n* = 1 Cervical spinal cord, *n* = 2 sensorimotor polyneuropathy (TN, MN, SN, and FN)	*n* = 2 paraesthesia, *n* = 1 weakness, *n* = 1 gait difficulties, *n* = 2 voiding difficulty	Both patient's symptoms partially improved after N_2_O cessation and supplementation therapy
Neveu J et al 2019^13^	France	15 F	Sensorimotor polyneuropathy (PN, TN)	Gait difficulties, weakness, areflexia & paraesthesia	1‐year follow‐up persisting mild symptoms of polyneuropathy
Middleton JA & Roffers JA 2017^14^	USA	22 M	Sensorimotor polyneuropathy (upper and lower limbs)	Weakness, foot drop, & paraesthesia	5 months complete resolution of the upper extremity but continued residual weakness in the right ankle
Van den Hoven C et al 2022^15^	Belgium	35 Male	Cervical and thoracic spinal cord, demyelinating & axonal sensorimotor polyneuropathy (lower limbs)	Numbness, gait difficulty & abdominal discomfort	Not reported
Vael L et al 2021^16^	Belgium	30 Male	Cervical spinal cord, axonal motor polyneuropathy	Paraesthesia, weakness & gait disturbances	Neurological symptoms rapidly decreased within a few days
Padayachee Y et al 2021^17^	UK	19 Male	Cervical and thoracic spinal cord	Paraesthesia, weakness and ataxia	Clinical improvements after supplement therapy and N_2_O abstinence.
Dong X et al 2019^18^	China	22 Male	Cervical spinal cord, axonal motor polyneuropathy	Quadriplegia, urinary incontinence and constipation	Discharged after 3 months with no obvious difficulties walking
Chen H & Huang C 2016^19^	Taiwan	20 Female	Cervical spinal cord, sensorimotor polyneuropathy (MN, UN, TN, PN, and SN)	gait difficulty, paraesthesia	At 3 weeks, with rehab, patient could walk independently
Sleeman I et al 2016^20^	UK	29 Female	cervical and thoracic spinal cord	Numbness, gait difficulty, paraesthesia & polyuria	2 years later, the patient could mobilize with the aid of crutches, though still liable to falls
Safari A et al 2013^21^	Iran	50 Male	cervical spinal cord	Ataxia, paraesthesia	At 4 weeks, patient's neurological symptoms improved after supplement treatment
Al‐Sadawi M et al 2018^22^	USA	22 Male	cervical spinal cord	Paraesthesia, weakness & gait difficulty	At 6 weeks, the patient admitted to continued use of N_2_O, & complained of paraesthesia
Zhang J et al 2021^23^	China	16 F, 18 F, 19 F, 20 F, 22 F, 22 F, 22 F, 24 F, 24 F, 17 M, 21 M, 22 M, 23 M, 24 M, 27 M, 28 M, 29 M, 31 M, 32 M & 35 M	Cervical *n* = 15, thoracic *n* = 3 & lumbar *n* = 1 spinal cord, *n* = 12 axonal motor polyneuropathy, *n* = 4 demyelination motor polyneuropathy	Numbness *n* = 18, weakness *n* = 15 & gait difficulty *n* = 4	16 patients recovered in 0.5–3.5 months and one patient gait difficulty and suffered from numbness and memory loss
Strauss J & Qadri SF 2021^24^	USA	45 Male	PN neuropathy	Numbness & gait difficulty	Full resolution after 24 weeks
Marotta DA & Kesserwani H 2020^25^	USA	41 Male	cervical spinal cord	Paraesthesia	At 2 weeks, patient's neurological symptoms improved after supplement treatment
Yuan JL et al 2017^26^	China	20 Female	cervical and thoracic spinal cord, sensorimotor polyneuropathy	Paraesthesia & gait difficulty	At 3 months, patient's neurological symptoms improved after supplement treatment and cessation of N_2_O
Hu M et al 2014^27^	Taiwan	16 Female	cervical and thoracic spinal cord, mild polyneuropathy	Numbness, gait difficulty & weakness	At 1 month after supplement treatment, the ataxia and numbness had improved
Ghobrial M et al 2012^28^	USA	19 Male	cervical spinal cord	Numbness & weakness	The patient's symptoms resolved entirely over 48 h
Srichawla BS 2022^29^	USA	44 Male	thoracic spinal cord	Rigidity, gait difficulty & memory loss	No improvement of neurological symptoms after 10 days of supplement treatment
Campdesuner V et al 2020^30^	USA	42 Female	cervical spinal cord	Headache, speech difficulty, numbness and gait difficulty	The patient's neurological symptoms improved after supplement treatment
Mancke F et al 2016^31^	Germany	35 Male	cervical spinal cord	Numbness, paraesthesia and gait difficulty	At 6‐month, substantial symptom improvement after supplement therapy and N_2_O abstinence
Agarwal P et al 2021^32^	USA	19 Male	cervical spinal cord	Paraesthesia and ataxia	The patient's neurological symptoms improved during hospitalization after supplement treatment
Morris N et al 2014^33^	USA	22 Male	Motor polyneuropathy (TN, PN)	Gait difficulty, numbness	At 7‐month, substantial symptom improvement after supplement therapy and N_2_O abstinence.
Cheng HM et al 2013^34^	Australia	22 Female	Cervical spinal cord	Paraesthesia, gait difficulty	At 18‐months, substantial symptom improvement after supplement therapy
Sluyts Y et al 2021^35^	Belgium	19 M, 18 M, 30 M, 19F, 18F, 22 M, 24F, 23 M	*n* = 3 axonal sensorimotor polyneuropathy, *n* = 1 sensory neuropathy (TN), mild motor neuropathy, *n* = 6 cervical spinal cord	*n* = 5 gait difficulty, *n* = 5 numbness, *n* = 3 paraesthesia and *n* = 1 weakness	*n* = 5 symptom improvement after supplement therapy, *n* = 3 not reported
Buizer A et al 2017^36^	Netherlands	31 Male	cervical spinal cord	Paraesthesia, weakness	At 3 months, neurological substantial symptom improvement after supplement therapy and N_2_O abstinence
Thompson AG et al 2015^37^	UK	22 M, 27 M, 23 F	*n* = 1 Polyneuropathy, *n* = 1 demyelinating neuropathy with additional axonal loss	*n* = 2 numbness, paraesthesia, gait difficulty, weakness	*n* = 2 at 6 months some symptom improvement after supplement therapy, *n* = 1 substantial symptom improvement after supplement therapy

Abbreviations: F, female; M, male; MN, median nerve; PN, peripheral nerve; SN, sural nerve; TN, tibial nerve; UN, ulnar nerve..

## 4 INTERPRETATION

**FIGURE 3:**
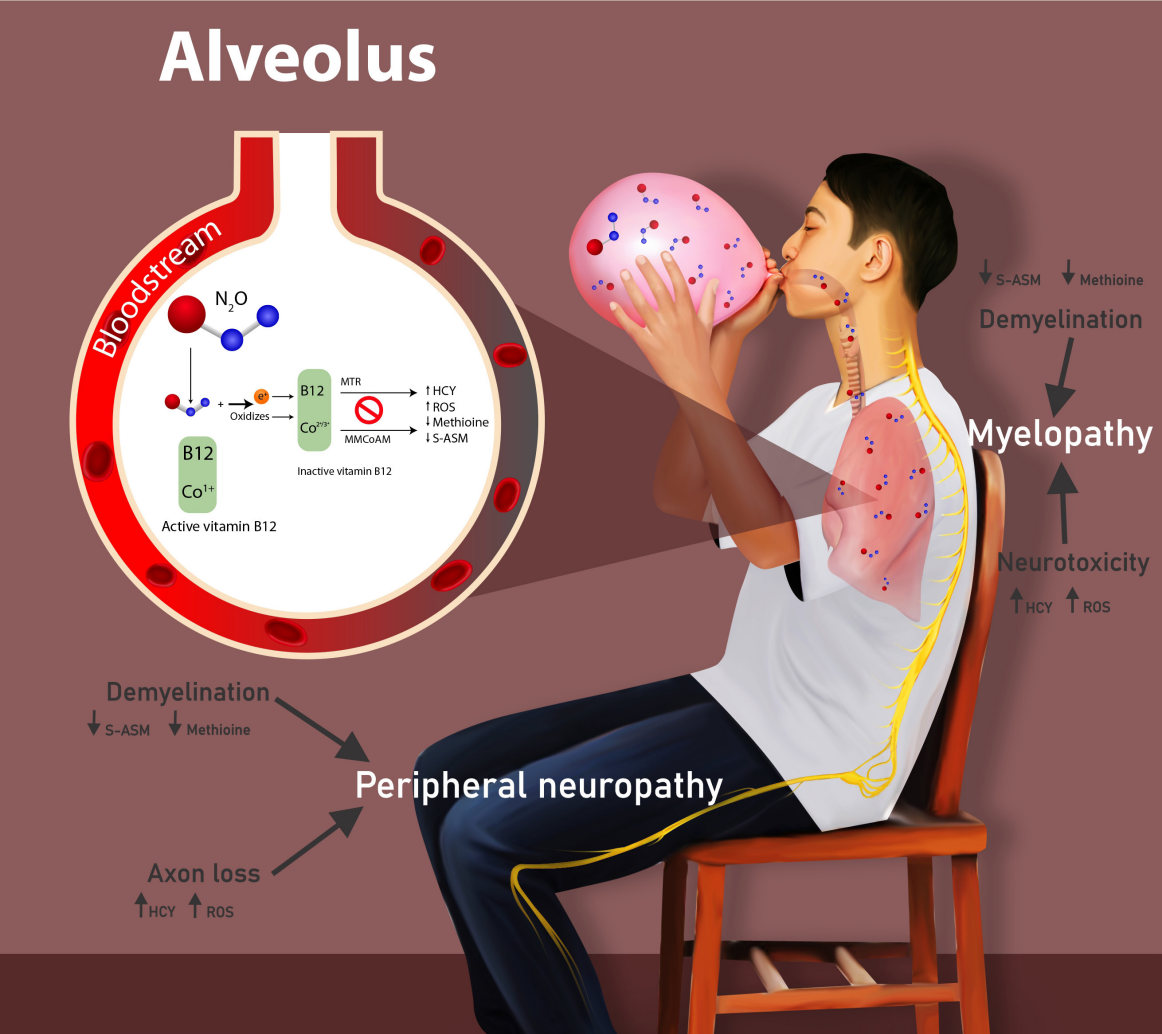
Illustration to demonstrate consumption of N_2_O and the chemical interactions that cause vitamin B_12_ deficiency. This results in demyelination and axonal loss in the central and peripheral nervous systems, and subsequent myelopathy and sensorimotor polyneuropathy, respectively; HCY, homocysteine; MMCoAM, methylmalonyl CoA mutase; MTR, methionine synthase; ROS, reactive oxygen species; S‐ASM, S‐ Adenosylmethionine.

Vitamin B_12_, otherwise known as cobalamin, is a water‐soluble vitamin, which is important in the development and myelination of axons in the nervous system, healthy red blood cell formation, and deoxyribonucleic acid (DNA) synthesis.^3^ This vitamin can be low in the following groups: pernicious anemia (often in older patients), gastrointestinal disorders (such as Crohn's and celiac disease), vegetarians, and when using certain drugs such as metformin.^4^ However, it can also be critically low from N_2_O abuse, as is documented in this case study and those included in our literature review (Table 3). In the plasma, vitamin B_12_ is bound to two types of proteins, these are holotranscobalamin and holohaptocorrin. The former is the active form of cobalamin and is important in the uptake of vitamin B_12_ into cells and tissues. Cobalamin has two major forms that are needed in two metabolic pathways: adenosyl cobalamin and methyl cobalamin. The former (adenosyl cobalamin) plays a major role in converting methylmalonyl‐CoA to succinyl‐CoA, this is important in the formation of myelin sheath proteins (Figure 3). The latter (methyl cobalamin) converts homocysteine to methionine, a process important in DNA synthesis and thus axon integrity.^5^, ^6^ N_2_O inhibits this action by oxidizing the cobalt ion (Co^+^) in cobalamin from its monovalent, active cobalt form to the inactive, bivalent cobalt (Co^2+^) or trivalent (Co^3+^) form (Figure 3), thus inactivating methionine synthetase. Consequently, this prevents the production of methionine from homocysteine, causing macrocytic anemia, as seen in this case. In addition, N_2_O interferes with the methylation of methylmalonyl‐CoA to succinyl‐CoA and thus myelin proteins cannot be methylated causing axonal swelling and eventual axonal loss.^7^ As is seen in the current case, the patient has a severe generalized sensorimotor polyneuropathy (Tables 1 and 2), which was seen in 53% of the 96 cases in our literature review, no doubt caused by the above pathophysiological process.

In this case report, the cervical MRI showed an increased T2 signal in the posterior columns at C2 and C3 consistent with subacute combined spinal cord degeneration, while the literature review reported that 52 of the 96 patients suffered cervical myelopathy. The main neuropathological changes in the affected spinal cord often include initial swelling of the myelin sheath surrounding axons, which has been documented to be reversible, followed by demyelination and axon loss, which is irreversible.^6^ From a regional stand point, this seems to primarily affect the posterior columns, but also the postero‐lateral regions of the cord.^8^ It has also been suggested that there are inhibitory effects on N‐methyl‐d‐asparate receptors in the central nervous system, stimulation of noradrenergic pathways and sympathetic actions via α‐1‐adrenergic stimulation, although these are not well understood.^8^, ^9^

Treatments will begin with ceasing N_2_O use and intramuscular vitamin B_12_ injections. Approximately 75% of the 96 patients in the review saw either a reduction or complete resolution of neurological systems if they followed this treatment over subsequent weeks. Exogenous methionine will also provide a direct substrate for methionine synthase, while the body is in the early stages of replacing the inactive form of vitamin B_12_ as well as additional multivitamin daily.^8^, ^10^ To address the bilateral foot drop, physiotherapy and a rehabilitation program would assist with balance and joint mobility, possibly with the use of ankle‐foot orthotics, as well as occupational therapy assessment regarding mobility and to mitigate any fall risks. Moreover, periodic monitoring of vitamin B_12_ levels, as well as repeated cervical MRI and repeated nerve‐conduction studies would allow for progressive quantification of the myelopathy and polyneuropathy, respectively.

## 5 CONCLUSION

In summary, we have reported a unique case of cervical myelopathy and generalized sensorimotor polyneuropathy, and a systematic literature review, to highlight the dangers of recreational N_2_O abuse. Given how cheap and readily available N_2_O is and that recreational drug users consider it a harmless alternative to other illicit drugs, it is important that users be alerted and educated about the serious and long‐term neurological consequences of even relatively short‐term abuse of this substance. While severe vitamin B_12_ deficiency can cause subacute combined spinal cord degeneration, with UMN dysfunction, it is important to acknowledge the role vitamin B_12_ has on peripheral nerve function also, which accounts for the patient's LMN signs and severe sensorimotor polyneuropathy.

## AUTHOR CONTRIBUTIONS

**Ventzi Bonev:** Conceptualization; data curation; formal analysis; project administration; supervision; validation; writing – review and editing. **Mark Wyatt:** Conceptualization; data curation; formal analysis; project administration; resources; software; validation; visualization; writing – original draft; writing – review and editing. **Matthew Barton:** Formal analysis; project administration; resources; validation; visualization; writing – original draft; writing – review and editing. **Michael Leitch:** Conceptualization; data curation; formal analysis; funding acquisition; investigation; methodology; project administration; resources; software; supervision; validation; visualization; writing – original draft; writing – review and editing.

## FUNDING INFORMATION

None.

## CONFLICT OF INTEREST

None declared; the authors whose names are listed immediately below certify that they have NO affiliations with or involvement in any organization or entity with any financial interest (such as honoraria; educational grants; participation in speakers' bureaus; membership, employment, consultancies, stock ownership, or other equity interest; and expert testimony or patent‐licensing arrangements), or non‐financial interest (such as personal or professional relationships, affiliations, knowledge, or beliefs) in the subject matter or materials discussed in this manuscript.

## CONSENT

Written informed consent was obtained from the patient to publish this report in accordance with the journal's patient consent policy.

## Data Availability

Data sharing not applicable to this article as no datasets were generated or analysed during the current study.

## References

[1] Redmond J , Cruse B , Kiers L . Nitrous oxide‐induced neurological disorders: an increasing public health concern. Intern Med J. 2022;52(5):740‐744. 34569693 10.1111/imj.15544

[2] Kaar SJ , Ferris J , Waldron J , Devaney M , Ramsey J , Winstock A . Up: the rise of nitrous oxide abuse. An international survey of contemporary nitrous oxide use. J Psychopharmacol. 2016;30(4):395‐401. 26912510 10.1177/0269881116632375

[3] Li Y , Dong J , Xu R , et al. Clinical epidemiological characteristics of nitrous oxide abusers: a single‐center experience in a hospital in China. Brain Behav. 2021;11:e2416. 34775689 10.1002/brb3.2416 PMC8671768

[4] Hirvioja J , Joutsa J , Wahlsten P , Korpela J . Recurrent paraparesis and death of a patient with ‘whippet’ abuse. Oxf Med Case Reports. 2016;3:41‐43. 10.1093/omcr/omw012 PMC4794556 26989492

[5] Smith CM , McCann P , Slauer R , Gilbert EB . Recreational nitrous oxide and pernicious anemia–associated vitamin B_12_ deficiency in a patient presenting with sensorimotor polyneuropathy. Prim Care Companion CNS Disord. 2021;23(2):28458. 10.4088/PCC.20l02751 34000117

[6] Einsiedler M , Voulleminot P , Demuth S , et al. A rise in cases of nitrous oxide abuse: neurological complications and biological findings. J Neurol. 2022;269(2):577‐582. 34245346 10.1007/s00415-021-10702-7 PMC8272450

[7] Zhao B , Zhao L , Li Z , Zhao R . Subacute combined degeneration induced by nitrous oxide inhalation: two case reports. Medicine. 2020;99(18):e19926. 32358361 10.1097/MD.0000000000019926 PMC7440322

[8] Berling E , Fargeot G , Aure K , et al. Nitrous oxide‐induced predominantly motor neuropathies: a follow‐up study. J Neurol. 2022;269(5):2720‐2726. 34741241 10.1007/s00415-021-10858-2

[9] Porruvecchio E , Shrestha S , Khuu B , et al. Functional vitamin B12 deficiency in association with nitrous oxide inhalation. Cureus. 2022;14(1):e21394. 35103219 10.7759/cureus.21394 PMC8776518

[10] Choi C , Kim T , Park KD , Lim OK , Lee JK . Subacute combined degeneration caused by nitrous oxide intoxication: a report of two cases. Ann Rehabil Med. 2019;43(4):530‐540. 31499607 10.5535/arm.2019.43.4.530 PMC6734019

[11] Beal JC , Cheng Y , Merchant S , Zarnegar R . An acute severe sensorimotor polyneuropathy in the setting of nitrous oxide abuse. Neurohospitalist. 2020;10:293‐296. 32983349 10.1177/1941874420910648 PMC7495705

[12] Hsu CK , Chen YQ , Lung VZ , His SC , Lo HC , Shyu HY . Myelopathy and polyneuropathy caused by nitrous oxide toxicity: a case report. Am J Emerg Med. 2012;30(6):1016.e3‐1016.e6. 10.1016/j.ajem.2011.05.001 22169583

[13] Neveu J , Perelman S , Suisse G , Monpoux F . Severe hyperhomocysteinemia and peripheral neuropathy as side effects of nitrous oxide in two patients with sickle cell disease. Arch Pediatr. 2019;26(7):419‐421. 31630905 10.1016/j.arcped.2019.09.006

[14] Middleton JA , Roffers JA . Peripheral neuropathy due to recreational use of nitrous oxide presenting after an ankle sprain with foot drop. Orthopedics. 2018;41(3):432‐433. 10.3928/01477447-20171102-05 29120005

[15] van den Hoven C , Lambrechts S , Reynders T . Neuro‐image: nitrous oxide‐induced myelopathy due to vitamin B12 deficiency. Acta Neurol Belg. 2022;122(1):203‐205. 33598882 10.1007/s13760-021-01616-2

[16] Vael L , Özsarlak Ö . MRI of nitrous oxide‐related subacute cervical myelopathy. J Belg Soc Radiol. 2021;105(1):22. 33870086 10.5334/jbsr.2347 PMC8034397

[17] Padayachee Y , Richards C , Morgan O . Inhaled nitrous oxide‐induced functional B12 deficiency. BMJ Case Rep. 2021;14(3):e240447. 10.1136/bcr-2020-240447 PMC7959227 33722915

[18] Dong X , Ba F , Wang R , Zheng D . Imaging appearance of myelopathy secondary to nitrous oxide abuse: a case report and review of the literature. Int J Neurosci. 2019;129(3):225‐229. 30234413 10.1080/00207454.2018.1526801

[19] Chen HJ , Huang CS . Nitrous oxide‐induced subacute combined degeneration presenting with dystonia and pseudoathetosis: a case report. Acta Neurol Taiwan. 2016;25(2):50‐55. 27854092

[20] Sleeman I , Wiblin L , Burn D . An unusual cause of falls in a young woman. J R Coll Physicians Edinb. 2016;46(3):160‐162. 27959349 10.4997/JRCPE.2016.304

[21] Safari A , Emadi F , Jamali E , Borhani‐Haghighi A . Clinical and MRI manifestations of nitrous oxide induced vitamin B12 deficiency: a case report. Iran J Neurol. 2013;12(3):111‐113. 24250916 PMC3829298

[22] Al‐Sadawi M , Claris H , Archie C , Jayarangaiah A , Oluya M , McFarlane SI . Inhaled nitrous oxide ‘whip‐its!’ causing subacute combined degeneration of spinal cord. Am J Med Case Rep. 2018;6(12):237‐240.31058215

[23] Zhang J , Xie D , Zou Y , et al. Key characteristics of nitrous oxide‐induced neurological disorders and differences between populations. Front Neurol. 2021;27(12):627183. 10.3389/fneur.2021.627183 PMC8110825 33986715

[24] Strauss J , Qadri SF . Myelopathy secondary to vitamin b12 deficiency induced by nitrous oxide abuse. Cureus. 2021;13(10):e18644. 34786239 10.7759/cureus.18644 PMC8580124

[25] Marotta DA , Kesserwani H . Nitrous oxide induced posterior cord myelopathy: beware of the methyl folate trap. Cureus. 2020;12(7):e9319. 32850197 10.7759/cureus.9319 PMC7444745

[26] Yuan JL , Wang SK , Jiang T , Hu WL . Nitrous oxide induced subacute combined degeneration with longitudinally extensive myelopathy with inverted V‐sign on spinal MRI: a case report and literature review. BMC Neurol. 2017;17(1):1‐4. 29282001 10.1186/s12883-017-0990-3 PMC5745895

[27] Hu MH , Huang GS , Wu CT , Hung PC . Nitrous oxide myelopathy in a pediatric patient. Pediatr Emerg Care. 2014;30(4):266‐267. 24694883 10.1097/PEC.0000000000000110

[28] Ghobrial GM , Dalyai R , Flanders AE , Harrop J . Nitrous oxide myelopathy posing as spinal cord injury: case report. J Neurosurg Spine. 2012;16(5):489‐491. 22385084 10.3171/2012.2.SPINE11532

[29] Srichawla BS . Nitrous oxide/Whippits‐induced thoracic spinal cord myelopathy and cognitive decline with Normal serum vitamin B₁₂. Cureus. 2022;14(4):e24581. 35664396 10.7759/cureus.24581 PMC9148417

[30] Campdesuner V , Teklie Y , Alkayali T , Pierce D , George J . Nitrous oxide‐induced vitamin B12 deficiency resulting in myelopathy. Cureus. 2020;12(7):e9088. 32685323 10.7759/cureus.9088 PMC7366039

[31] Mancke F , Kaklauskaitė G , Kollmer J , Weiler M . Psychiatric comorbidities in a young man with subacute myelopathy induced by abusive nitrous oxide consumption: a case report. Subst Abuse Rehabil. 2016;7:155‐159. 27729826 10.2147/SAR.S114404 PMC5047713

[32] Agarwal P , Khor SY , Do S , Charles L , Tikaria R . Recreational nitrous oxide‐induced subacute combined degeneration of the spinal cord. Cureus. 2021;13(11):e19377. 34909324 10.7759/cureus.19377 PMC8653952

[33] Morris N , Lynch K , Greenberg SA . Severe motor neuropathy or neuronopathy due to nitrous oxide toxicity after correction of vitamin B12 deficiency. Muscle Nerve. 2015;51(4):614‐616. 25297001 10.1002/mus.24482

[34] Cheng HM , Park JH , Hernstadt D . Subacute combined degeneration of the spinal cord following recreational nitrous oxide use. BMJ Case Rep. 2013;2013:bcr2012008509. 10.1136/bcr-2012-008509 PMC3618752 23476009

[35] Sluyts Y , Vanherpe P , Amir R , Vanhoenacker F , Vermeersch P . Vitamin B12 deficiency in the setting of nitrous oxide abuse: diagnostic challenges and treatment options in patients presenting with subacute neurological complications. Acta Clin Belg. 2022;23:1‐7. 10.1080/17843286.2021.2015555 34886750

[36] Buizert A , Sharma R , Koppen H . When the laughing stops: subacute combined spinal cord degeneration caused by laughing gas use. J Addict Med. 2017;11(3):235‐236. 28166085 10.1097/ADM.0000000000000295

[37] Thompson AG , Leite MI , Lunn MP , Bennett DL . Whippits, nitrous oxide and the dangers of legal highs. Pract Neurol. 2015;15(3):207‐209. 25977272 10.1136/practneurol-2014-001071 PMC4453489

